# Are the self-stigma and perceived stigma of patients treated with methadone or buprenorphine still a problem fifty years after the marketing authorization for opioid agonist treatment? The observational STIGMA study

**DOI:** 10.1186/s13722-024-00506-1

**Published:** 2024-10-16

**Authors:** Mélanie Pinhal, Benoit Schreck, Juliette Leboucher, STIGMA-group, Benoit Schreck, Julie Abesdris, Malcolm Barrangou-Pouyes-Darlas, Emeline Eyzop, Valentine Galantai, Lucie Robert Kunze-, Sylvain Lambert, Stéphane Prétagut, Audrey Verholleman, Caroline Victorri-Vigneau, Edouard-Jules Laforgue, Marie Grall-Bronnec

**Affiliations:** 1 Addictive Medicine and Psychiatry Department, Nantes Université, CHU Nantes, 44000 Nantes, France; 2 Nantes Université, Univ Tours, CHU Nantes, CHU Tours, INSERM, MethodS in Patients Centered Outcomes and HEalth ResEarch, SPHERE, 44000 Nantes, France; 3 Pharmacology Department, Nantes Université, CHU Nantes, 44000 Nantes, France; 4 Addiction Medicine and Psychiatry Department, Saint Jacques Hospital, 85, Rue Saint Jacques, 44093 Nantes Cedex 1, France

**Keywords:** Opioid use disorder, Opioid agonist treatment, Barrier to treatment, Self-stigma, Perceived stigma

## Abstract

**Background:**

In the context of the opioid overdose crisis, understanding the barriers to seeking, attaining and remaining in treatment for patients with opioid use disorder (OUD) is a public health issue. To date, very few studies have assessed the “self-stigma” (i.e., the internalization of negative societal attitudes and stereotypes about oneself, leading to self-judgment) and “perceived stigma” (i.e., the belief that others hold negative attitudes towards oneself due to a particular condition) experienced by patients with OUD receiving opioid agonist treatment (OAT), and none have done so in France. Our study aimed to quantify self-stigma, explore some aspects of perceived stigma, determine the factors associated with greater self-stigma and examine whether the level of self-stigma was related to a delay in seeking care.

**Methods:**

The STIGMA study was a monocentric, cross-sectional study. The data were collected in a French hospital addiction medicine department. Participants were outpatients with current or past OUD who were still receiving or had received OAT. A questionnaire assessing sociodemographics; OUD characteristics; perceived stigma; and quantification of self-stigma by the Self-Stigma Scale-Short, was administered.

**Results:**

A total of 73 questionnaires were included in the analysis. Nearly two-thirds of the patients had a “moderate to high” level of self-stigma**.** These patients were significantly younger at OUD onset and were significantly more likely to have at least one dependent child than patients reporting a “very low to low” level of self-stigma. Nearly half of the participants experienced perceived stigma from a healthcare professional regarding their OUD or OAT, and nearly one-third of the participants were refused care from a healthcare professional because of their OUD or OAT. Moreover, a quarter of the sample reported delaying care due to fear of being stigmatized. We did not find a relationship between self-stigma levels and a delay in seeking care.

**Conclusions:**

Our study highlights the need to detect stigma and to improve training in addiction medicine.

## Background

More than 20 years ago, the world experienced a resurgence in the consumption of heroin and, more broadly, opioids. This was particularly evident in the USA due to new requirements for pain assessment and the widespread prescription of opioid analgesics in the 2000s [1]. Globally, opioid use disorder (OUD) affects more than 16 million people, and OUD-related harms are manifold, including a high risk of morbidity and mortality [2], as well as professional, financial, and legal consequences. This makes OUD a major public health concern [3, 4].

Treatment of patients with OUD involves a biopsychosocial model of care that incorporates psychosocial interventions in addition to approved medications [5]. Opioid agonist treatment (OAT), such as methadone and buprenorphine, is currently the evidence-based pharmacological treatment for OUD. Despite initial controversies, the clinical efficacy of these medications has been demonstrated by numerous studies worldwide: the outcomes include reduced all-cause mortality, increased life expectancy of persons who use drugs, decreased withdrawal symptoms, reduced cravings, decreased illicit opioid consumption, the cessation of intravenous drug use, and decreased transmission of infectious agents [4–8]. Patients also experience benefits such as improved quality of life [9–12], improved employment rates and social well-being [13], and improved family stability [14].

In France, buprenorphine is accessible (initiation of treatment, prescription renewals) in primary care, whereas methadone must be introduced in specialized centers, with a possible continuation in primary care, usually once the dosage is stabilized. According to the 2020 French Monitoring Centre for Drugs and Drug Addiction report, about 210,000 individuals regularly used opioids in 2017. Among them, ~ 180,000 were receiving OAT, with 162,000 in general practice and 18,000 in specialized centers [15]. However, despite a significant increase in the number of patients with OUD who are managed in general practice, the provision of care remains heterogeneous. In France in 2019, 26% of OAT prescribers in general practice were treating 75% of the patients [16].

Various studies show the persistence of certain misconceptions that act as barriers to care [17, 18]. Gradually, the term *"addictophobia"* has emerged in the grey literature, referring to the hostility of some healthcare professionals toward persons who use drugs, leading to discriminatory attitudes [19]. All these factors contribute to the emergence of *“public stigma” (or “social stigma”)*, which is defined by the discrimination of one group by another, in accordance with negative societal stereotypes [20, 21]. Perceptions of this public stigma could be experienced by an individual, leading to the *“perceived stigma”*, defined as the belief that others hold negative attitudes towards oneself due to a particular condition [22]. Public and perceived stigma are the most prominent forms of stigma [23]. However, the harm caused by stigma is not simply the direct result of discrimination by others but can result from the internalization of the attitudes and beliefs of the public by a stigmatized person [24]. *“Internalized stigma”*, or *“self-stigma*”, can be described as the acceptance and integration of societal prejudices and their application to oneself or the group to which one belongs [24–28]. Self-sigma is the consequence of public stigma and perceived stigma [29], and it seems to best reflect patients' suffering and the clinical consequences of this phenomenon [30]. Self-stigma negatively impacts patient hope, self-esteem, empowerment, therapeutic adherence, and psychiatric symptoms [26, 31, 32]. It also leads to a delay in seeking care [33] and affects all psychosocial variables, including social integration and quality of life [26, 34, 35].

Several studies have explored stigma toward patients with OUD or people who use drugs more broadly [36–38]. Outside the healthcare system, patients with OUD experience significant social rejection, leading to difficulties in accessing employment and housing, as well as legal issues [21]. Among healthcare professionals, stigma is particularly observed in general practitioners, paramedical staff in general [39–41], pharmacists (for example, those who refuse to provide syringe kits that allow patients who inject opioids to reduce the risk of interpersonal transmission of infectious diseases like HIV and hepatitis, as well as injection site complications) [42], and hospital services [43]. It would be intuitive to think that patients who are participating in OAT programs, without the status of “addicts” or “drug users”, would no longer be stigmatized. However, medical intervention does not always reduce stigma [44]. Thus, studies conducted in the USA and Europe among patients receiving OAT who no longer used illegal opioids revealed persistent public and perceived stigma in the medical, social, family, professional, legal, political, and administrative domains [40, 45–48]. To use the example of pharmacists again, some refuse to dispense OAT, mistakenly perceiving it as equivalent to dispensing a "legalized form" of an illegal drug, or believing that using the treatment is merely a “transfer” of the addiction [49].

Exploring this phenomenon is a major public health challenge given the multifaceted and disastrous consequences of stigma on recovery, as reported by a recent qualitative study conducted in persons who were in recovery from OUD or a family member of someone with OUD [21]. To our knowledge, very few studies worldwide, and no studies in France, have assessed perceived stigma and self-stigma of patients with OUD receiving OAT. Therefore, we decided to fill this gap by conducting a study that aimed to quantify self-stigma in these patients. The secondary objectives were to explore some aspects of perceived stigma, determine the factors associated with greater self-stigma, and examine whether the level of self-stigma was related to a delay in seeking care.

## Methods

### Study setting and participants

The STIGMA study was a monocentric, observational, cross-sectional study conducted in a population of patients with current or past OUD (we did not seek to differentiate between patients with current or past OUD, as the study focused on the possible experience of stigmatization. Even if the OUD was currently resolved, everyone could recall situations of perceived stigma or self-stigma). These patients also had to be receiving or have received OAT and had to be currently followed or previously followed at the Addiction Medicine Department of Nantes University Hospital (France) since 2012. Patients who did not understand spoken or written French, who lacked social security coverage, or who were under legal protection were not eligible for the study.

Eligible patients were offered the opportunity to complete an anonymous self-administered questionnaire. Patients still under follow-up in the department were systematically invited to participate during a medical consultation. If they agreed, they completed the questionnaire on-site. Patients no longer under follow-up were invited to participate through a telephone call and later by mail. They received the questionnaire at home, completed it, and returned it using a prepaid envelope. In cases of missing or inconsistent data, the affected patients were recontacted by phone or questioned following a medical consultation, or their medical records were reviewed, in order to complete or correct the information.

As our study was exploratory, we aimed to include as many patients as possible during the period from November 16, 2022, to April 6, 2023, without a predefined calculation of the necessary sample size.

### Measures

The self-administered questionnaire, built by the first (general practitioner) and last (psychiatrist specializing in addiction) authors, consisted of four parts: sociodemographic data; OUD characteristics; perceived stigma; self-stigma.

#### Sociodemographic data

We collected data on the following variables: sex, age, educational level (highest obtained degree), employment status, type of income, the presence of indebtedness, housing situation, marital status, and the presence of a dependent child, drug-using spouse and drug-using friends.

#### OUD characteristics

We collected data on the following variables: age at first opioid use, age at onset of OUD (defined as the age of daily illegal or nonmedical opioid use), and the time between the onset of OUD and the start of the first OAT sequence. Finally, we asked for details about the different OATs received throughout the treatment course, including the age of onset, current OAT status, and opioid consumption in the last 2 months.

The questionnaire assessed the presence of negative consequences of OUD, including secondary psychiatric and somatic conditions and socioaffective, professional, financial, or legal harm.

#### Measurement of perceived stigma

We developed a questionnaire (6 questions with binary yes/no answers) for the purpose of this study to explore some aspects of perceived stigma. The questions addressed: the quality of the relationship with the general practitioner; the presence of at least one perceived stigmatizing attitude from a healthcare professional (medical and paramedical) regarding the patient’s OUD or OAT; the existence of at least one instance of refusal of care by a healthcare professional because of the patient’s OUD or OAT; and the presence of consciously delayed healthcare seeking due to fear of being stigmatized because of OAT use. This questionnaire has not been pre-tested.

#### Measurement of self-stigma

We used the Self-Stigma Scale-Short (SSS-S), the French and simplified version of the Self-Stigma Scale (SSS), which has been validated for use in patients with psychiatric disorders [50, 51]. The scale consisted of 9 statements for which patients must indicate their degree of agreement or disagreement on a Likert scale ranging from 1 (strongly disagree) to 5 (strongly agree). The total score ranges from 9 to 45. A higher score indicates greater self-stigma, and there are five categories for results: class 1 (very low stigma: scores from 9 to 15 points); class 2 (low stigma: scores from 16 to 22 points); class 3 (average stigma: scores from 23 to 33 points); class 4 (high stigma: scores from 34 to 42 points); and class 5 (very high stigma: scores from 43 to 45 points). The SSS-S has good internal validity, and the internal consistency coefficients for different scores range from satisfactory to excellent. Participants in this study were asked to answer by considering themselves as “patients receiving OAT (current or past)”.

### Statistical analyses

First, continuous variables (age, age of first opioid use, age of onset of OUD, time between the onset of OUD and the start of the first OAT sequence) are described by their means and standard deviations, and categorical variables (other variables in Table 1) are described by numbers and percentages.

**Table 1:** Description of the study sample and comparison of the patients according to their self-stigma level (n = 73)

	Global sample (n = 73^a^)	“Very low to low self-stigma” (n = 28^a^)	“Moderate to high self-stigma” (n = 45^a^)	p value
	Mean ± sd or n (%)			
*Sociodemographic characteristics*				
Sex (men)	53 (72.6)	23 (82.1)	30 (66.7)	0.149
Age (in years)	41.3 ± 9.62	43.8 ± 9.63	39.7 ± 9.38	0.113
Educational level (≥ 12 years)	40 (56.3) [N = 71]	16 (59.3) [N = 27]	24 (54.5) [N = 44]	0.697
Employment status (being active)	28 (38.4)	10 (35.7)	18 (40.0)	0.714
Regular income	37 (551.4) [N = 72]	14 (50)	23 (52.3) [N = 44]	0.851
Debt	21 (28.8)	10 (35.7)	11 (24.4)	0.301
Stable housing	58 (79.5)	23 (82.1)	35 (77.8)	0.654
Marital status (life as a couple)	29 (39.7)	8 (28.6)	21 (46.7)	0.124
≥ 1 Dependent child	23 (31.5)	4 (14.3)	19 (42.2)	**0.012**
Drug-using spouse	9 (12.3)	2 (7.1)	7 (15.6)	0.288
Drug-using friends	42 (57.5)	16 (57.1)	26 (57.8)	0.957
*OUD characteristics*				
Age at first opioid use (in years)	21.5 (6.0)	22.4 ± 6.64	21.0 ± 5.57	0.314
Age at onset of OUD (in years)	23.7 (6.41)	25.5 ± 6.99	22.7 ± 5.85	**0.031**
Time between the onset of OUD and the start of the first OAT sequence	5.12 (3,97)	5.7 ± 4.17	4.7 ± 3.85	0.348
OAT	73 (100)			
- Past - Current	9 (12.3) 64 (87.7)	3 (10.7) 25 (89.3)	6 (13.3) 39 (86.7)	0.741
Current OAT	64 (87.7)			
- Current methadone use - Current buprenorphine use	46 (63.0) 18 (24.7)	18 (64.3) 7 (25.0)	28 (62.2) 11 (24.4)	0.935
Opioid use during the previous two last months	23 (31.9) [N = 72]	9 (32.1)	14 (31.8) [N = 44]	0.977
*OUD negative consequences*				
Psychiatric	61 (83.6)	22 (78.6)	39 (86.7)	0.364
Somatic	24 (32.9)	9 (32.1)	15 (33.3)	0.916
Socioaffective	42 (57.5)	16 (57.1)	26 (57.8)	0.957
Professional	45 (62.5) [N = 72]	15 (53.6)	30 (68.2) [N = 44]	0.212
Legal	30 (41.1)	9 (32.1)	21 (46.7)	0.220
Financial	34 (46.6)	14 (50.0)	20 (44.4)	0.644
*Perceived stigma*				
≥ 1 Stigmatizing attitude from a healthcare professional regarding OUD	41 (57.7) [N = 71]	15 (53.6)	26 (60.5) [N = 43]	0.565
≥ 1 Stigmatizing attitude from a healthcare professional regarding OAT	31 (43.1) [N = 72]	10 (35.7)	21 (47.7) [N = 44]	0.316
≥ 1 Refusal of care by a healthcare professional because of OUD	21 (30.4) [N = 69]	9 (33.3) [N = 27]	12 (28.6) [N = 42]	0.675
≥ 1 Refusal of care by a healthcare professional because of OAT	24 (33.8) [N = 71]	10 (35.7)	14 (32.6) [N = 43]	0.784
Delayed health care seeking due to fear of being stigmatized because of OAT use	18 (25) [N = 72]	5 (17.9)	13 (29.5) [N = 44]	0.264

Bold significance of *p* value < 0.05

^a^Corresponds to the headcount for each variable unless otherwise indicated

Second, we explored factors associated with greater self-stigma. We conducted bivariate analysis, comparing the characteristics of patients with SSS-S scores less than 23 (“very low to low self-stigma” group) to those with SSS-S scores greater than or equal to 23 (“moderate to very high self-stigma” group). We deemed the clinical and functional impact on patients to be significant from a medium level of self-stigma onward, which justified our choice. The chi-square test and Mann‒Whitney test were used to test the independence of variables. We considered a p value < 0.05 as the threshold for significance. As the study was purely exploratory, no adjustments were made to account for alpha inflation.

Finally, we assessed the strength of the relationship between the SSS-S score and the time between the onset of OUD and the first treatment sequence using Spearman's correlation.

### Ethics

The STIGMA study was conducted in accordance with the Good Clinical Practice Guidelines and the Declaration of Helsinki and was approved by a local medical ethics committee (GNEDS: Nantes ethics group in the health field) on 19/09/2022. As part of noninterventional research, inclusion was performed through an oral recording of nonopposition from each patient. A copy of the information letter was then given to the patient, and a dated copy signed by the physician was retained in the department.

## Results

### Description of the sample

Of the 232 patients potentially eligible for the study, 75 completed the questionnaire. Two questionnaires were excluded due to missing data on self-stigma status. Ultimately, 73 questionnaires were included in the analysis. The sociodemographic and OUD characteristics of the sample are presented in Table 1.

Nearly half of the participants experienced a perceived stigmatizing attitude from a healthcare professional regarding their OUD or OAT, and nearly one-third of the participants reported being refused care from a healthcare professional because of their OUD or OAT. Moreover, a quarter of the sample reported delaying care due to fear of being stigmatized because of their OAT. The detailed results are presented in Table 1. However, the majority of patients (84.9%) described their relationship with their general practitioner as a good or moderate quality, while 12.3% reported having a poor-quality relationship or no general practitioner at all.

Self-stigma, as assessed by the patients, was perceived as moderate, with a mean SSS-S score of 24.7 ± 7.5. Table 2 indicates the distribution of the participants according to their SSS-S score. Twenty-eight patients (38.4% of the sample) had a score lower than 23, indicating a low to very low level of self-stigma. Forty-five patients (61.6% of the sample) had a score between 23 and 42, indicating a moderate to high level of self-stigma. No patients had a very high level of self-stigma (i.e., a score higher than 42).

**Table 2:** Distribution of the patients according to their self-stigma level (N = 73)

	Class 1: very low	Class 2: low	Class 3: moderate	Class 4: high	Class 5: very high
Number of patients (percentage)	9 (12.3)	19 (26.0)	37 (50.7)	8 (11.0)	0

### Comparison of the patients according to their self-stigma level

Since no patient reported a very high level of self-stigma, we renamed the second group: “moderate to high self-stigma”. As shown in Table 1, only two variables were significantly different between the “very low to low self-stigma” group and the “moderate to high self-stigma” group. Patients with “moderate to high self-stigma” were younger at OUD onset and were more likely to have at least one dependent child.

### Relationship between self-stigma and the time between the onset of OUD and the start of the first treatment sequence (which represents the delay of seeking care)

**Fig. 1:**
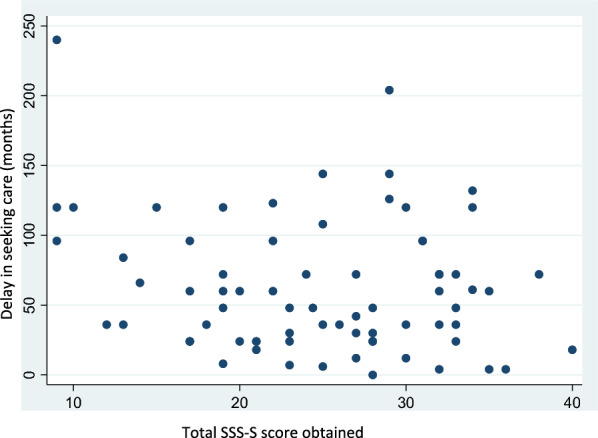
Scatter plot showing the relationship between the delay in seeking care (in months) and the SSS-S score obtained

Figure 1 depicts the distribution of time delays in seeking care (in months) based on the obtained SSS-S score. The Spearman correlation coefficient was 0.11 and was not statistically significant (p = 0.376). Therefore, we could not conclude that an association exists between these two variables.

## Discussion

### Main results

This retrospective study focused on patients with (current or past) OUD who were currently receiving or had received OAT. In particular, we investigated self-stigma and perceived stigma. Several key findings of this work should be highlighted.

First, our results indicate a moderate level of self-stigma related to current or past OAT use, with a mean SSS-S score of 24.7/45, corresponding to moderately high self-stigma. This result is consistent with previous studies exploring self-stigma in patients receiving OAT, although these studies were conducted in different cultural contexts or used different questionnaires [40, 46]. Given that nearly 30 years have passed since the marketing authorization of OAT in France, these results seem contradictory to the substantial knowledge we currently have about these treatments. Over the past three decades, the effectiveness and benefits of OAT have been well-documented, and, in theory, healthcare professionals and students should have received extensive training on this subject. Therefore, we expected to find a lower level of stigmatization. This suggests that training may be insufficient or inconsistent among healthcare professionals. Additionally, we can hypothesize that some older patients may have experienced stigma in the past, before improvements in professional training, and may still internalize these biases. However, our study did not test association between the total duration of OAT or age and self-stigma. As mentioned in the background, public stigma can also manifest at social, professional, and familial levels, which might contribute to maintaining a high levels of self-stigma despite advances in scientific knowledge about OAT [52].

Second, nearly half of our patients perceived stigma from a healthcare professional due to their OAT, with a greater proportion perceiving stigma related to their OUD. Again, these results could be explained by insufficient training of French healthcare professionals in the management of patients with OUD and in the prescription of OAT. Elsewhere in the world, several authors supported this conclusion when they argued that stigmatizing attitudes toward patients with OUD did not cease after recovery or entry into treatment [53] or highlighted discrimination in pharmacies [54]. However, our figures are higher than those reported by Frischknecht et al. [46], as only 7% of "opioid-dependent" patients in their sample reported feeling stigmatized within the healthcare system. This difference may be explained by the methodology used. In our study, patients were asked to indicate if they had experienced at least one stigmatizing attitude from a healthcare professional (event if it was single), whereas in Frischknecht’s study, patients indicated in which way they felt discriminated (in general). Furthermore, approximately one-third of the sample in our study was affected by refusals of care from a healthcare professional. The study by Kimmel et al. [43], among others, also mentioned refusals of care: 15.1% of analyzed refusals of care in hospitals were related to OUD or OAT, with 7.8% were directly related to OAT and 7.3% were related to OUD. Several qualitative studies conducted with general practitioners have attempted to explain these difficulties in caring for patients with OUD who are receiving OAT. General practitioners highlighted the time-consuming follow-up of patients with OUD, their mistrust of the patients with OUD, the risk of misuse/resale of OAT, and their difficulties regarding a lack of training or accessing specialists and healthcare networks [55, 56]. Moreover, the gradual increase in “medical deserts” in France in recent years may have increased these refusals of care due to the “congestion” of consultations in primary care settings [57].

Third, we identified two significant differences between patients according to their level of self-stigma. On the one hand, patients with “moderate to high” self-stigma more frequently had at least one dependent child. We can assume that during prenatal and postnatal consultations or during follow-up consultations for the child, stigma may have been perceived by parents with OUD who were receiving OAT, who then internalized this stigma. Several studies have mentioned this stigma during pregnancy [58, 59]. Healthcare professionals may be uncomfortable with continuing OAT for future mothers, even though evidence-based clinical guidelines encourage the prescription of OAT during pregnancy [59]. Another stigmatizing attitude involves being reluctant to prescribe additional analgesics or anxiolytics after childbirth, likely due to concerns about potential misuse of these medications by the mother [59]. On the other hand, patients with “moderate to high” self-stigma were younger at the onset of OUD. To our knowledge, no data have been found in the literature regarding this possible association. As we did not observe a difference in the age at first opioid use, we can hypothesize that these patients had greater vulnerability to developing OUD (low self-esteem, anxiety or depressive symptoms, etc.), which in turn could lead to self-stigma. Another explanation is that patients who were younger at the onset of OUD had higher levels of self-stigma because they perceived the stigma at an age when they were more likely to internalize it. Nonetheless, there was no difference between the two groups regarding the delay in seeking care, and there was no correlation between the level of self-stigma and the delay in seeking care.

### Strengths and limitations of the study

These results must be viewed within the context of several limitations. The main limitation of this work is undoubtedly the small number of included patients (participation rate of 32.3%), leading to a lack of power. Moreover, as this study was exploratory, we cannot affirm that the observed differences are not due to chance. Regarding biases, there is, *first*, a selection bias inherent in the sample recruitment method. The included patients may have been more motivated because they were concerned about the subject. Conversely, we can also assume that the patients who did not respond were those with a higher level of self-stigma. Internalized stigma would be part of the denial in recognizing this stigma, which would lead patients to not feel concerned about this problem and to not complete the questionnaire. Furthermore, the significant number of nonresponses from patients no longer followed in the service requires cautious interpretation, and if the nonresponding patients actually differed from those who responded, this could introduce a nonresponse bias. Additionally, recruiting patients who were exclusively followed or who had been followed in the Addiction Medicine Department of a university hospital increased the probability of including patients with more severe addiction issues or complex biopsychosocial problems, probably adversely influencing the average SSS-S score obtained. Finally, we deliberately chose not to differentiate between patients who still had OUD and those who were in recovery. This may have influenced the retrospective assessment of their lived experience. *Second*, the use of a custom-made questionnaire to evaluate perceived stigma, which had not been previously validated, may also have introduced a measurement bias. *Third*, there was initially a significant amount of missing or inconsistent data in the questionnaires, partly explained by recall bias, particularly concerning the age at which the first sequence of OAT began (i.e., missing at random). We minimized this limitation as much as possible by completing or correcting the data according to the established procedure (in the worst case, there were missing responses from 4 patients). Finally, the last limitation arose from the choice of the assessment method (i.e., a short, self-administered questionnaire, without open-ended fields where patients could describe the experienced stigma situations), mainly driven by the need to minimize the duration of the evaluation and to improve participation in the study: neither the severity of OUD nor the main route of opioid administration was assessed, while these clinical data may have influenced the level of stigma.

However, these limitations are compensated for by the strengths of the study. The sociodemographic characteristics of our sample patients are consistent with those observed in the literature, including the sex ratio, the average age of patients receiving OAT and the proportion of patients treated with methadone and buprenorphine in the Addiction Treatment Centers and specialized centers in France [60]. The external validity of this study is good because the sample is representative of the target population. Our results can, with caution, be generalized to patients who receive OAT in specialized settings in France. Furthermore, extending the study to patients no longer followed in the Addiction Medicine Department of the university hospital at the time of inclusion allows the results to be generalized, to some extent, to patients followed in outpatient settings who are initially followed in specialized settings. The real strength of our study is that it addresses an issue that has been little (or not at all) explored in France since the marketing authorization of OAT. Research has focused on the biopsychosocial outcomes of patients after the introduction of OAT, without addressing the issue of stigma. Additionally, this work directly encouraged patients to evaluate their feelings. Our study thus highlights certain existing gaps in the literature, where in the era of a “patient-centered approach”, most studies address the feelings of healthcare professionals and not the patients in question. Moreover, the main outcome measure was based on responses to a validated questionnaire with good psychometric properties, providing quantitative data, which is uncommon for such a topic explored mostly by qualitative methods [21]. Our study therefore complements those already carried out.

### Implications

Several implications arise from this work, the first being related to care. Our study highlights a tool for the rapid detection of self-stigma that is easy to use and reproducible in any patient who receives OAT; this tool can be used for screening purposes or to open a dialog.

Second, there is a need for better training in addiction medicine for all healthcare professionals to update knowledge, correct misconceptions, dismantle prejudices and the negative image of OAT, and improve prescribing skills to eliminate barriers to the proper care of patients benefiting from these treatments. Better training regarding addiction is particularly needed in the fields of maternal and pediatric health, and better training for addiction specialists in the follow-up of pregnant women in the context of OUD or the prescription of OAT are also desirable. We should also encourage interprofessional collaboration to support, among others, frontline general practitioners in managing patients receiving OAT, in order to reduce the number of refusals of care that may result from a lack of knowledge and a sense of isolation.

Third, several implications for research were identified. It would be necessary to replicate the study with a larger number of patients to identify different risk factors for OAT-related self-stigma and to reliably generalize the results to the entire French territory. This work could include patients followed in hospitals, those followed in Addiction Treatment Centers, and those followed in general practice. Moreover, it seems pertinent to complement our study with qualitative research with patients. This could explore OUD patients’ feelings and their perspectives on the subject, the different areas/places in daily life where they experience stigma regarding OAT, how to understand the adaptation mechanisms implemented to cope with stigma, and how to consider strategies to reduce this phenomenon or at least improve their care.

Finally, interventions aimed at reducing public and structural stigma (within various institutions) would be necessary to reduce self-stigmatization regarding OAT. This could include information and awareness campaigns targeted at the general population, for example. Besides we believe that the issues raised by our study deserve attention in other countries as well, where there are strict and harmful OAT restrictions.

## Data Availability

The datasets generated and analysed during the current study are not publicly available because data generated included sensitive data according to the French Data Protection Authority (CNIL), that could not be transferred to other researchers to guarantee participants’ anonymity. But they are available from the corresponding author on reasonable request.

## Abbreviations

OAT: Opioid agonist treatment
OUD: Opioid use disorder

## References

[1] Soelberg CD, Brown REJ, Du Vivier D, Meyer JE, Ramachandran BK. The US opioid crisis: current federal and state legal issues. Anesth Analg. 2017;125(5):1675–81. 29049113 10.1213/ANE.0000000000002403

[2] Dydyk AM, Jain NK, Gupta M. Opioid use disorder. In: StatPearls. Treasure Island (FL): StatPearls Publishing; 2024. Disponible sur: http://www.ncbi.nlm.nih.gov/books/NBK553166/. 31985959

[3] Paulozzi LJ, Zhang K, Jones CM, Mack KA. Risk of adverse health outcomes with increasing duration and regularity of opioid therapy. J Am Board Fam Med. 2014;27(3):329–38. 24808111 10.3122/jabfm.2014.03.130290 PMC6660001

[4] Santo T, Clark B, Hickman M, Grebely J, Campbell G, Sordo L, et al. Association of opioid agonist treatment with all-cause mortality and specific causes of death among people with opioid dependence. JAMA Psychiat. 2021;78(9):1–15. 10.1001/jamapsychiatry.2021.0976 PMC8173472 34076676

[5] ASAM Provider Guide—National Practice Guideline for the Treatment of Opioid Use Disorder—2020 Update. Disponible sur: https://eguideline.guidelinecentral.com/i/1224390-national-practice-guideline-for-the-treatment-of-opioid-use-disorder-2020-update/5?.

[6] Ma J, Bao YP, Wang RJ, Su MF, Liu MX, Li JQ, et al. Effects of medication-assisted treatment on mortality among opioids users: a systematic review and meta-analysis. Mol Psychiatry. 2019;24(12):1868–83. 29934549 10.1038/s41380-018-0094-5

[7] Kelty E, Hulse G, Joyce D, Preen DB. Impact of pharmacological treatments for opioid use disorder on mortality. CNS Drugs. 2020;34(6):629–42. 32215842 10.1007/s40263-020-00719-3

[8] MacArthur GJ, van Velzen E, Palmateer N, Kimber J, Pharris A, Hope V, et al. Interventions to prevent HIV and Hepatitis C in people who inject drugs: a review of reviews to assess evidence of effectiveness. Int J Drug Policy. 2014;25(1):34–52. 23973009 10.1016/j.drugpo.2013.07.001

[9] Feelemyer JP, Jarlais DCD, Arasteh K, Phillips BW, Hagan H. Changes in quality of life (WHOQOL-BREF) and addiction severity index (ASI) among participants in opioid substitution treatment (OST) in low and middle income countries: an international systematic review. Drug Alcohol Depend. 2014;134:251–8. 24200104 10.1016/j.drugalcdep.2013.10.011 PMC3880839

[10] Nosyk B, Guh DP, Sun H, Oviedo-Joekes E, Brissette S, Marsh DC, et al. Health related quality of life trajectories of patients in opioid substitution treatment. Drug Alcohol Depend. 2011;118(2–3):259–64. 21546173 10.1016/j.drugalcdep.2011.04.003

[11] Jalali A, Ryan DA, Jeng PJ, McCollister KE, Leff JA, Lee JD, et al. Health-related quality of life and opioid use disorder pharmacotherapy: a secondary analysis of a clinical trial. Drug Alcohol Depend. 2020;215:108221. 32777692 10.1016/j.drugalcdep.2020.108221 PMC7502461

[12] Aas CF, Vold JH, Skurtveit S, Lim AG, Ruths S, Islam K, et al. Health-related quality of life of long-term patients receiving opioid agonist therapy: a nested prospective cohort study in Norway. Subst Abuse Treat Prev Policy. 2020;15(1):68. 32883319 10.1186/s13011-020-00309-y PMC7469909

[13] Sun HM, Li XY, Chow EPF, Li T, Xian Y, Lu YH, et al. Methadone maintenance treatment programme reduces criminal activity and improves social well-being of drug users in China: a systematic review and meta-analysis. BMJ Open. 2015;5(1):e005997. 10.1136/bmjopen-2014-005997 PMC4289728 25573521

[14] Reno RR, Aiken LS. Life activities and life quality of heroin addicts in and out of methadone treatment. Int J Addict. 1993;28(3):211–32. 8440536 10.3109/10826089309039624

[15] Tableau de bord annuel des traitements de substitution aux opiacés—OFDT. Disponible sur: https://www.ofdt.fr/statistiques-et-infographie/tableau-de-bord-annuel-des-traitements-de-substitution-aux-opiaces/.

[16] Slama MR. Rôle des médecins généralistes dans la prescription des traitements de substitution aux opiacés chez les patients dépendants [Thèse d’exercice]. [France]: Université Paris 13; 2019.

[17] Bell J. Quality improvement for methadone maintenance treatment. Subst Use Misuse. 2000;35(12–14):1735–56. 11138706 10.3109/10826080009148239

[18] Pasman E, Lee G, Kollin R, Rodriguez B, Agius E, Madden EF, et al. Attitudes toward medication for opioid use disorder among substance use treatment providers. Subst Use Misuse. 2022;57(12):1828–36. 36041008 10.1080/10826084.2022.2115853

[19] Blansfield HN. Addictophobia. Conn Med. 1991;55(6):361. 1935058

[20] Krajewski C, Burazeri G, Brand H. Self-stigma, perceived discrimination and empowerment among people with a mental illness in six countries: Pan European stigma study. Psychiatry Res. 2013;210(3):1136–46. 23998361 10.1016/j.psychres.2013.08.013

[21] Judd H, Yaugher AC, O’Shay S, Meier CL. Understanding stigma through the lived experiences of people with opioid use disorder. Drug Alcohol Depend. 2023;249:110873. 37390780 10.1016/j.drugalcdep.2023.110873

[22] Chan KKS, Fung WTW, Leung DCK, Tsui JKC. The impact of perceived and internalised stigma on clinical and functional recovery among people with mental illness. Health Soc Care Community. 2022;30(6):e6102–11. 36254881 10.1111/hsc.14047

[23] Corrigan PW, Rao D. On the self-stigma of mental illness: stages, disclosure, and strategies for change. Can J Psychiatry. 2012;57(8):464–9. 22854028 10.1177/070674371205700804 PMC3610943

[24] Lauber C. Stigma and discrimination against people with mental illness: a critical appraisal. Epidemiol Psichiatr Soc. 2008;17(1):10–3. 18444451

[25] Ritsher JB, Phelan JC. Internalized stigma predicts erosion of morale among psychiatric outpatients. Psychiatry Res. 2004;129(3):257–65. 15661319 10.1016/j.psychres.2004.08.003

[26] Livingston JD, Boyd JE. Correlates and consequences of internalized stigma for people living with mental illness: a systematic review and meta-analysis. Soc Sci Med. 2010;71(12):2150–61. 21051128 10.1016/j.socscimed.2010.09.030

[27] Corrigan PW, Watson AC, Barr L. The self-stigma of mental illness: implications for self-esteem and self-efficacy. J Soc Clin Psychol. 2006;25(8):875–84.

[28] Corrigan PW, Watson AC. The paradox of self-stigma and mental illness. Clin Psychol Sci Pract. 2002;9(1):35–53.

[29] Evans-Lacko S, Brohan E, Mojtabai R, Thornicroft G. Association between public views of mental illness and self-stigma among individuals with mental illness in 14 European countries. Psychol Med. 2012;42(8):1741–52. 22085422 10.1017/S0033291711002558

[30] Vogel DL, Wade NG, Hackler AH. Perceived public stigma and the willingness to seek counseling: the mediating roles of self-stigma and attitudes toward counseling. J Couns Psychol. 2007;54:40–50.

[31] Link BG, Struening EL, Neese-Todd S, Asmussen S, Phelan JC. Stigma as a barrier to recovery: the consequences of stigma for the self-esteem of people with mental illnesses. Psychiatr Serv déc. 2001;52(12):1621–6. 10.1176/appi.ps.52.12.1621 11726753

[32] Corrigan PW, Larson JE, Rüsch N. Self-stigma and the «why try» effect: impact on life goals and evidence-based practices. World Psychiatry. 2009;8(2):75–81. 19516923 10.1002/j.2051-5545.2009.tb00218.x PMC2694098

[33] Jensen LF, Pedersen AF, Bech BH, Andersen B, Vedsted P. Psychiatric morbidity and non-participation in breast cancer screening. Breast. 2016;25:38–44. 26585065 10.1016/j.breast.2015.10.002

[34] Rosenfield S. Labeling mental illness: the effects of received services and perceived stigma on life satisfaction. Am Sociol Rev. 1997;62(4):660–72.

[35] Reinhard MA, Dewald-Kaufmann J, Wüstenberg T, Musil R, Barton BB, Jobst A, et al. The vicious circle of social exclusion and psychopathology: a systematic review of experimental ostracism research in psychiatric disorders. Eur Arch Psychiatry Clin Neurosci. 2020;270(5):521–32. 31586242 10.1007/s00406-019-01074-1

[36] Link BG, Phelan JC, Bresnahan M, Stueve A, Pescosolido BA. Public conceptions of mental illness: labels, causes, dangerousness, and social distance. Am J Public Health. 1999;89(9):1328–33. 10474548 10.2105/ajph.89.9.1328 PMC1508784

[37] Palamar JJ, Kiang MV, Halkitis PN. Predictors of stigmatization towards use of various illicit drugs among emerging adults. J Psychoactive Drugs. 2012;44(3):243–51. 23061324 10.1080/02791072.2012.703510

[38] Barry CL, McGinty EE, Pescosolido BA, Goldman HH. Stigma, discrimination, treatment effectiveness, and policy: public views about drug addiction and mental illness. Psychiatr Serv. 2014;65(10):1269–72. 25270497 10.1176/appi.ps.201400140 PMC4285770

[39] Paschkis Z, Potter ML. CE: acute pain management for inpatients with opioid use disorder. AJN Am J Nurs. 2015;115(9):24–32. 10.1097/01.NAJ.0000471243.30951.92 26273927

[40] Etesam F, Assarian F, Hosseini H, Ghoreishi F. Stigma and its determinants among male drug dependents receiving methadone maintenance treatment. Arch Iran Med. 2014;17:108–14. 24527971

[41] Fontesse S, Rimez X, Maurage P. Stigmatization and dehumanization perceptions towards psychiatric patients among nurses: a path-analysis approach. Arch Psychiatr Nurs. 2021;35(2):153–61. 33781393 10.1016/j.apnu.2020.12.005

[42] Paquette CE, Syvertsen JL, Pollini RA. Stigma at every turn: health services experiences among people who inject drugs. Int J Drug Policy. 2018;57:104–10. 29715589 10.1016/j.drugpo.2018.04.004 PMC5994194

[43] Kimmel SD, Rosenmoss S, Bearnot B, Larochelle M, Walley AY. Rejection of patients with opioid use disorder referred for post-acute medical care before and after an anti-discrimination settlement in Massachusetts. J Addict Med. 2021;15(1):20–6. 32675798 10.1097/ADM.0000000000000693 PMC7859880

[44] Pescosolido BA, Martin JK. The stigma complex. Ann Rev Sociol. 2015;41(1):87–116. 26855471 10.1146/annurev-soc-071312-145702 PMC4737963

[45] Lindgren BM, Eklund M, Melin Y, Graneheim UH. From resistance to existence—experiences of medication-assisted treatment as disclosed by people with opioid dependence. Issues Mental Health Nurs. 2015;36(12):963–70. 10.3109/01612840.2015.1074769 26735504

[46] Frischknecht U, Beckmann B, Heinrich M, Kniest A, Nakovics H, Kiefer F, et al. The vicious circle of perceived stigmatization, depressiveness, anxiety, and low quality of life in substituted heroin addicts. Eur Addict Res. 2011;17(5):241–9. 21654177 10.1159/000328637

[47] Luoma JB, Twohig MP, Waltz T, Hayes SC, Roget N, Padilla M, et al. An investigation of stigma in individuals receiving treatment for substance abuse. Addict Behav. 2007;32(7):1331–46. 17092656 10.1016/j.addbeh.2006.09.008

[48] Woods JS, Joseph H. Stigma from the viewpoint of the patient. J Addict Dis. 2015;34(2–3):238–47. 26076048 10.1080/10550887.2015.1059714

[49] Etcheverrigaray F, Bétaud C, Feuillet F, Grall-Bronnec M, Jolliet P, Victorri-Vigneau C. Pharmacists’ different profiles characterization about opioid substitution treatments. Therapie. 2016;71(4):379–87. 27203168 10.1016/j.therap.2016.01.008

[50] Golay P, Martinez D, Silva B, Morandi S, Bonsack C. Validation psychométrique d’une échelle française d’auto-stigmatisation auprès d’un échantillon de patients souffrant de troubles mentaux: la Self-Stigma Scale-Short (SSS-S). Annales Médico-psychologiques revue psychiatrique. 2022;180(9):899–904.

[51] King M, Dinos S, Shaw J, Watson R, Stevens S, Passetti F, et al. The Stigma Scale: development of a standardised measure of the stigma of mental illness. Br J Psychiatry. 2007;190:248–54. 17329746 10.1192/bjp.bp.106.024638

[52] McCradden MD, Vasileva D, Orchanian-Cheff A, Buchman DZ. Ambiguous identities of drugs and people: a scoping review of opioid-related stigma. Int J Drug Policy. 2019;74:205–15. 31671303 10.1016/j.drugpo.2019.10.005

[53] Garpenhag L, Dahlman D. Perceived healthcare stigma among patients in opioid substitution treatment: a qualitative study. Subst Abuse Treat Prev Policy. 2021;16:81. 34702338 10.1186/s13011-021-00417-3 PMC8549326

[54] Radley A, Melville K, Easton P, Williams B, Dillon JF. «Standing Outside the Junkie Door’-service users» experiences of using community pharmacies to access treatment for opioid dependency. J Public Health (Oxf). 2017;39(4):846–55. 27915259 10.1093/pubmed/fdw138

[55] Fraeyman J, Symons L, Van Royen P, Van Hal G, Peremans L. How to overcome hurdles in opiate substitution treatment? A qualitative study with general practitioners in Belgium. Eur J Gen Pract. 2016;22(2):134–40. 26799738 10.3109/13814788.2015.1120286

[56] Longman C, Temple-Smith M, Gilchrist G, Lintzeris N. Reluctant to train, reluctant to prescribe: barriers to general practitioner prescribing of opioid substitution therapy. Aust J Prim Health. 2012;18(4):346–51. 22950844 10.1071/PY11100

[57] Ministère du Travail, de la Santé et des Solidarités. Ministère du travail, de la santé et des solidarités. Lutter contre les déserts médicaux. Disponible sur: https://sante.gouv.fr/archives/masante2022/lutter-contre-les-deserts-medicaux/.

[58] Chandler A, Whittaker A, Cunningham-Burley S, Williams N, McGorm K, Mathews G. Substance, structure and stigma: parents in the UK accounting for opioid substitution therapy during the antenatal and postnatal periods. Int J Drug Policy. 2013;24(6):e35-42. 23688832 10.1016/j.drugpo.2013.04.004

[59] Kremer ME, Arora KS. Clinical, ethical, and legal considerations in pregnant women with opioid abuse. Obstetr Gynecol. 2015;126(3):474–8. 10.1097/AOG.0000000000000991 26244538

[60] Ndiaye A. Traitements de substitution aux opioïdes en France. Notes de l'OFDT, 4 pages, Mars 2023.ISBN : 979-10-92728-68-2. https://www.ofdt.fr/sites/ofdt/files/2023-08/field_media_document-3251-doc_num--explnum_id-33864-.pdf

